# Role of Telemedicine in Inflammatory Bowel Disease: Systematic Review and Meta-analysis of Randomized Controlled Trials

**DOI:** 10.2196/28978

**Published:** 2022-03-24

**Authors:** Lanlan Pang, Hengyu Liu, Zhidong Liu, Jinyu Tan, Long-yuan Zhou, Yun Qiu, Xiaoqing Lin, Jinshen He, Xuehua Li, Sinan Lin, Subrata Ghosh, Ren Mao, Minhu Chen

**Affiliations:** 1 Department of Gastroenterology The First Affiliated Hospital of Sun Yat-sen University Guangzhou China; 2 Zhongshan School of Medicine Sun Yat-sen University Guangzhou China; 3 Guangdong Provincial Key Laboratory of Orthopedics and Traumatology Department of Spine Surgery The First Affiliated Hospital of Sun Yat-sen University Guangzhou China; 4 Department of Radiology The First Affiliated Hospital of Sun Yat-sen University Guangzhou China; 5 NIHR Biomedical Research Centre Institute of Translational Medicine University Hospitals Birmingham NHS Foundation Trust Birmingham United Kingdom

**Keywords:** telemedicine, inflammatory bowel disease, quality of life, disease activity, mobile phone

## Abstract

**Background:**

Telemedicine plays an important role in the management of inflammatory bowel disease (IBD), particularly during a pandemic such as COVID-19. However, the effectiveness and efficiency of telemedicine in managing IBD are unclear.

**Objective:**

This systematic review and meta-analysis aimed to compare the impact of telemedicine with that of standard care on the management of IBD.

**Methods:**

We systematically searched the PubMed, Cochrane Library, EMBASE, Web of Science, and Scopus databases on April 22, 2020. Randomized controlled trials comparing telemedicine with standard care in patients with IBD were included, while conference abstracts, letters, reviews, laboratory studies, and case reports were excluded. The IBD-specific quality of life (QoL), disease activity, and remission rate in patients with IBD were assessed as primary outcomes, and the number of in-person clinic visits per patient, patient satisfaction, psychological outcome, and medication adherence were assessed as secondary outcomes. Review Manage 5.3 and Stata 15.1 were used for data analysis.

**Results:**

A total of 17 randomized controlled trials (2571 participants) were included in this meta-analysis. The telemedicine group had higher IBD-specific QoL than the standard care group (standard mean difference 0.18, 95% CI 0.01 to 0.34; *P*.03). The number of clinic visits per patient in the telemedicine group was significantly lower than that in the standard care group (standard mean difference −0.71, 95% CI −1.07 to −0.36; *P*<.001). Subgroup analysis showed that adolescents in the telemedicine group had significantly higher IBD-specific QoL than those in the standard care group (standard mean difference 0.42, 95% CI 0.15 to 0.69; I2=0; *P*.002), but there was no significant difference between adults in the 2 groups. There were no significant differences in disease activity, remission rate, patient satisfaction, depression, self-efficacy, generic QoL, and medication adherence outcomes between the telemedicine and standard care groups.

**Conclusions:**

Telemedicine intervention showed a promising role in improving IBD-specific QoL among adolescents and decreased the number of clinic visits among patients with IBD. Further research is warranted to identify the group of patients with IBD who would most benefit from telemedicine.

## Introduction

Inflammatory bowel disease (IBD), including Crohn disease and ulcerative colitis, is a group of chronic inflammatory disorders of the gut. The prevalence of IBD is increasing worldwide, with 3 million cases recorded in the United States in 2015 and 4 million cases projected in Canada by 2030 [1,2]. Because of its recurrent relapsing-remitting nature, IBD exerts a substantial economic and health burden on patients and their families, health organizations, and nations [3,4]. The lack of curative therapy for this condition entails lifelong medication and follow-up that need to be effectively monitored in patients with IBD [5].

Telemedicine was first defined by the World Health Organization as health care service provided to patients at a distance through information communication technologies (ie, SMS text messaging, web-based applications, real-time telephone) [6]. It is a broad term. Although the specific telemedicine subtypes (telemonitoring, tele-education, and teleconsulting) exhibit significant heterogeneity, they are closely tied together by the concept of remote health care resources delivery [7]. Given the convenience of communication technologies, clinicians have been increasingly using eHealth interventions as a supplementary tool to conduct follow-up and provide education, including disease status and medication instruction. Electronic medical technology has been proven to change the course of certain chronic diseases such as diabetes and asthma [8-11]. Patients with IBD, commonly diagnosed as having the condition at a young age and deemed to need lifelong follow-up for long-term remission, could also potentially benefit from telemedicine intervention for preventing disease progression and reducing complications and operation rates [12-14]. Telemedicine has played an important role in the management of IBD during the recent COVID-19 pandemic [15]. Specific tools such as the IBD Monitoring Index for Mobile Health have been developed and validated for clinical management [16-21]. Others tools such as the IBD disk have been adapted to smartphone apps to monitor IBD-associated disability [22,23].

However, there is no consensus on remote health care technology preferences for IBD management because of contradictory results and high heterogeneity among studies. Few studies precisely quantified the magnitude of intervention effects [24-26], although many studies demonstrated that telemedicine had a major impact on the management of IBD [7,27,28]. We aimed to estimate the differences between telemedicine and standard care in the management of IBD by conducting a systematic review and meta-analysis of randomized controlled trials (RCTs).

## Methods

This study was performed in accordance with the PRISMA (Preferred Reporting Items for Systematic Reviews and Meta-Analyses) guidelines.

### Search Strategy and Selection Criteria

#### Literature Search Strategy

Two investigators (LLP and ZDL) independently searched publications in the PubMed, Cochrane Library, EMBASE, Web of Science, and Scopus databases (search date April 22, 2020) using the following search terms: (telemedicine OR telemonitor OR e-health OR telehealth OR telecommunication OR telemanagement OR telecare OR (telephone monitoring) OR telenursing OR ((remote and short) message service) OR (mobile health) OR (mobile applications) OR teleconsultation) AND ((inflammatory bowel disease) OR (ulcerative colitis) OR (Crohn’s disease)). The search in the Web of Science, Cochrane Library, and Scopus Google Scholar databases was limited to titles and abstracts. However, no limitations were applied to the search of PubMed and EMBASE. We also manually searched the reference lists and related literature to identify additional publications. The data sets used in this study can be obtained from the corresponding author on request. Records were imported into EndNote X 9.0 software (Clarivate) to eliminate duplications.

#### Eligibility Criteria and Study Selection

Two authors (LLP and HYL) independently screened the titles, abstracts, and keywords of the identified articles and selected suitable papers for full review. Disagreements were resolved by a third investigator (ZDL) or by consensus.

The studies included had to meet the following PICOS (participants, interventions, control, outcomes, study design) criteria described in Textbox 1 [6].

The exclusion criteria were as follows: conference abstracts, letters, reviews, laboratory studies, and case reports in which the necessary information could not be extracted; non-English publications; and studies that did not report the outcomes required.

**Inclusion criteria.**

1. *P (participants)*

Patients diagnosed as having IBD

2.* I (interventions)*

Telemedicine defined as “the use of electronic information and communication technologies for the delivery of health care when there exist distances between patents and health care providers” such as internet, mobile phone applications, and SMS text messaging

3. *C (control)*

Standard care or usual care provided by the medical center according to IBD treatment guidelines

4. *O (outcomes)*

At least one of the following outcomes: inflammatory bowel disease–specific quality of life, disease activity, remission rate, generic quality of life, self-efficacy, depression, medication adherence, patient satisfaction, and the number of clinic visits per patient

5. *S (study design)*

Only randomized controlled trials

### Risk of Bias

Two reviewers (LLP and HYL) independently assessed the quality and risk of bias of the included studies using the Cochrane Handbook of Systematic Reviews of Interventions [29]. In addition, the revised Jadad scale was also applied to assess the quality of the included articles [30]. Any disagreement was resolved by the third reviewer (ZDL).

### Data Extraction

Two authors (LLP and HYL) independently extracted data, and disagreements were resolved by a third investigator (ZDL). Extracted data included first author, publication year, country, participant characteristics (age, gender, disease type, and disease activity status), intervention, follow-up time, and outcomes. The investigators contacted authors to obtain original data not reported in the published papers. If the number of telemedicine intervention groups was more than one, amalgamation of these groups was performed. If outcomes were reported more than once, the updated data would be evaluated on priority.

### Outcomes and Definitions

Primary outcomes in our study included IBD-specific QoL, disease activity, and remission rate. Secondary outcomes included generic QoL, self-efficacy, depression, medication adherence, patient satisfaction, and the number of in-person clinic visits per patient.

Except the number of clinic visits, reported outcomes were measured by specific questionnaires or scales. For instance, IBD-specific QoL was assessed by the IBD questionnaire (IBDQ) [31]. Disease activity was assessed by the Mayo score, Walmsley index, or Seo index for ulcerative colitis or indeterminate colitis and by the Harvey Bradshaw Index for Crohn disease [32-35]. Additionally, remission rate was defined as the proportion of patients in clinical remission at the endpoint or during the intervention [36,37]. Patient satisfaction was defined by scales (eg, consultation satisfaction questionnaire) evaluating the acceptance of care provided by clinical staff [38].

### Data Synthesis and Statistical Analysis

All data were analyzed using Review Manager 5.3 (The Cochrane Collaboration) and Stata 15.1 (StataCorp). We used standardized mean difference (SMD) with 95% CI to calculate continuous variables and relative risk with 95% CI to calculate discontinuous variables. Owing to the heterogeneity between the included studies, we used a random-effects model to assess a relatively more conservative estimate of the 95% CI. Heterogeneity was evaluated using the Cochrane *Q* statistic and *I^2^* statistic. Subgroup analysis was conducted if needed, focusing on predefined stratification including the follow-up time (<12 months or not) and patient characteristics (adults or adolescents or patients aged above 18 years or not). Funnel plots, Egger test, and Begg test were used to examine potential publication bias.

## Results

### Search Process, Study Characteristics, and Quality Assessment

**Figure 1:**
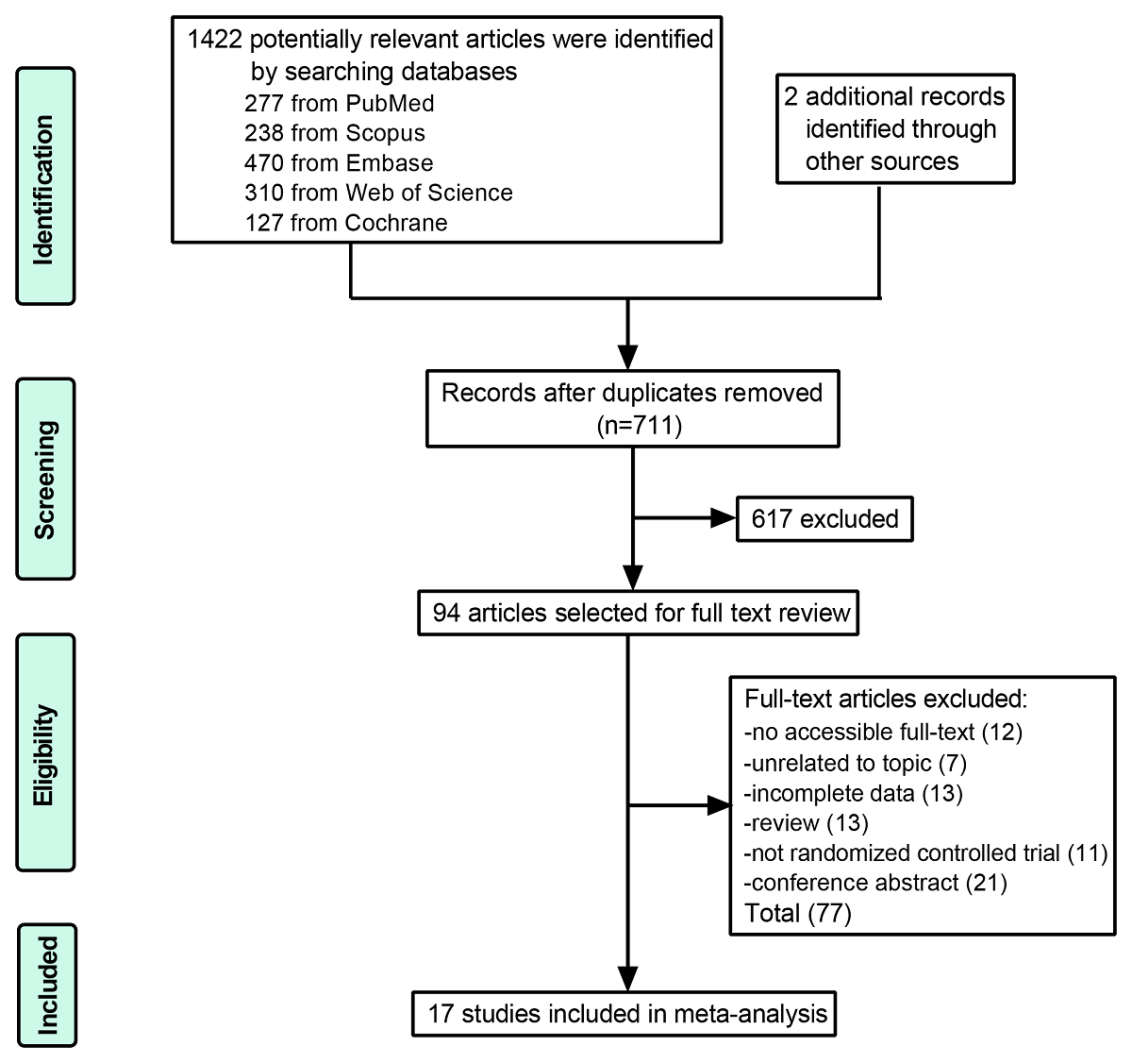
Flow diagram of the selection of enrolled studies.

A total of 1422 articles were identified after searching the databases, and 2 additional articles were included through search of the references. Of the 711 unique studies obtained after removing duplications, 617 irrelevant articles were eliminated and 94 were assessed in full text. The following publications were excluded: 12 articles because of a lack of accessible full text, 13 because of incomplete data, 7 for being unrelated to the topic, and 11 for failure of randomization; 21 conference abstracts; and 13 reviews. Finally, 17 RCTs were considered potentially eligible and comprised 2571 patients from 2010 to 2020; most of these RCTs were conducted in the United States [39-45], followed by the Netherlands [46-48], New Zealand [49,50], Denmark [12,51], the United Kingdom [52], Spain [53], Ireland [12], and Turkey [13]. The process of selecting enrolled studies is shown in Figure 1.

**Figure 2:**
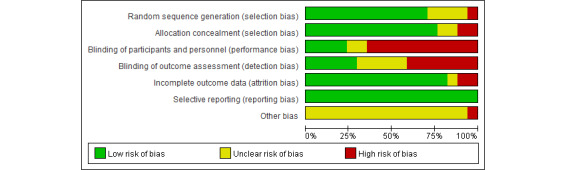
Risk of bias graph.

**Figure 3:**
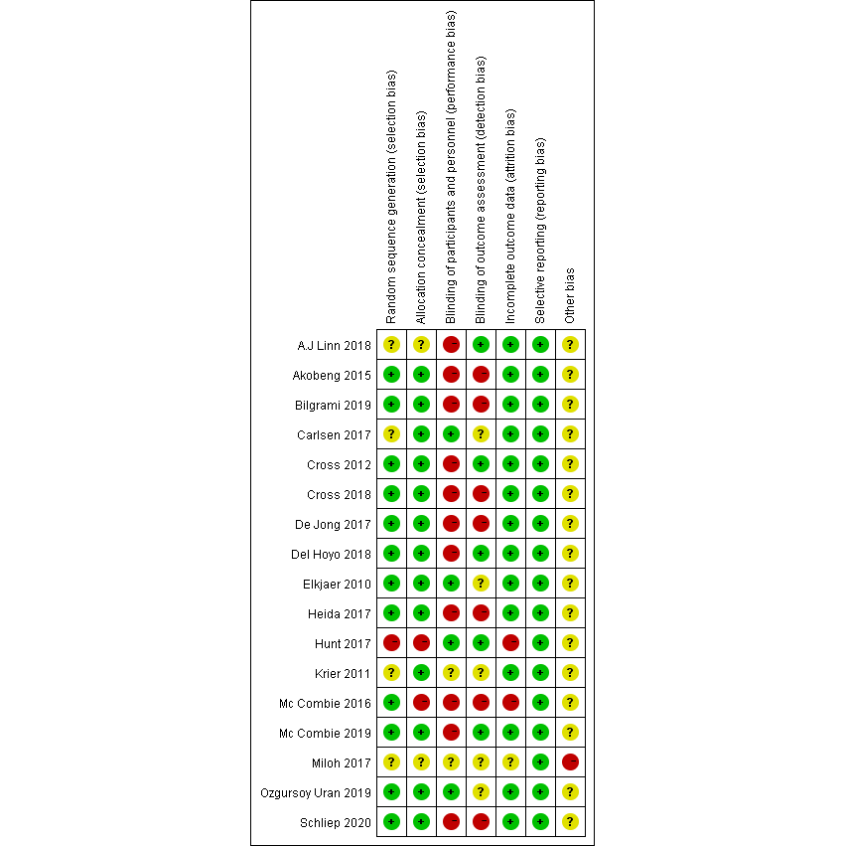
Risk of bias summary.

Table 1 summarizes the key characteristics of the included studies and participants. The results of the revised Jadad scale for the enrolled studies are also shown in Table 1 and indicate that 13 identified studies were of high quality (ranging from 5 to 7). Three of the included studies are from the same clinical trial but report different outcomes. The methodological quality of enrolled studies is shown in Figure 2 and Figure 3.

**Table 1:** Characteristics of the included studies and participants.

Reference	Country	Jadad score	Age (years), mean (SD)/mean (IQR)/mean (range)^a^	Male/total^a^ (%)	Participants ^a^, n	Participant characteristics	Intervention/application^a^	Follow-up time (months)	Outcomes
Linn et al, 2018 [48]	Netherlands	1+1+0+1=3	40.84 (14.51) vs 45.21(17.15)	40.4 vs 51.5	52 vs 33	Individuals diagnosed with IBD^b^ Individuals receiving immunosuppressant or biological therapy for the first time	Telemonitoring through web or SMS text messaging vs usual care	6	Patient satisfaction Self-efficacy Medication adherence score
Ako Beng et al, 2015 [52]	United Kingdom	2+2+0+1=5	13.9 (12.1,15.9) vs 13.8 (11.2,15.3)	68 vs 57	44 vs 42	Young people (aged 8-16 years) with IBD	Teleconsulting through telephone vs usual care	12	IBD-specific QoL^c^ Patient satisfaction
Bil Grami et al, 2019^d^ [42]	United States	2+2+0+1=5	39.7 (13) vs 37.7 (11.6) vs 40.2 (11)	38.5 vs 47.4 vs 36.9	75 vs 72 vs 75	Adults with IBD who experienced an IBD flare within 2 years prior to the trial Individuals at least 18 years of age	Telemonitoring and tele-education through mobile phone with SMS text messaging vs standard care	12	Self-efficacy
Carlsen, 2017 [51]	Denmark	1+2+1+1=5	15.1 (1.82) vs 14.7 (2.11)	37 vs 46	27 vs 26	Children and adolescents, 10-17 years old, diagnosed as having IBD	Telemonitoring through web-based applications, SMS text messaging, and phone call vs standard care	24	Number of clinic visits per patient
Cross et al, 2012 [39]	United States	2+2+0+1=5	41.7 (13.9) vs 40.3 (14.4)	40 vs 32	25 vs 22	Adults with ulcerative colitis	Telemonitoring through home unit-server PC provider vs standard care	12	IBD-specific QoL Disease activity Medication adherence rate
Cross, 2018^d^ [40]	United States	2+2+0+1=5	40.1 (13.2) vs 36.4 (11.5) vs 40.1 (11.7)	41.7 vs 43.1 vs 45.3	115 vs 116 vs 117	Adults ≥18 years of age diagnosed as having IBD who experienced at least one IBD flare in the 2 years prior to the baseline visit	Telemonitoring and tele-education through mobile phone with SMS text messaging vs standard care	12	IBD-specific QoL Disease activity Remission rate
De Jong, 2017[47]	Netherlands	2+2+0+1=5	44.0 (±14.1) vs 44.1 (14.2)	42 vs 41	465 vs 444	Outpatients aged 18-75 years with IBD and without an ileoanal or ileorectal pouch anastomosis	Telemonitoring through web-based applications on a tablet or smartphone vs standard care	12	IBD-specific QoL Number of outpatient visits per patient Medication adherence rate Self-efficacy
Del Hoyo et al, 2018 and 2019 [18,53]	Spain	2+2+0+1=5	41.32(19-66) vs 40.91(24-60) vs 39.31(22-61)	42.9 vs 57.1 vs 57.1	21 vs 21 vs 21	Adults ≥18 years of age diagnosed as having IBD for at least 6 months Patients who had complex IBD when immunosuppressants or biologic agents were initiated	Telemonitoring through a web-based system with smartphone apps or a tablet or through the telephone vs standard care	6	IBD-specific QoL Remission rate Disease activity Generic QoL Medication adherence score and rate Patient satisfaction
Elkjaer et al, 2010 [12]	Denmark and Ireland	2+2+2+1=7	Denmark: 40 (21-69) vs 44 (21-69) Ireland: 41 (18-66) vs 46 (19-65)	Denmark: 49.5 vs 31.1 Ireland: 60.8 vs 41.5	Denmark: 105 vs 106 Ireland: 51 vs 41	Patients aged 18-69 years who met the international diagnostic criteria for mild to moderate ulcerative colitis and were treated with 5-aminosalicylic acid	Tele-education through web-based applications vs usual care	12	Medication adherence rate Remission rate Number of clinic visits
Heida et al, 2017 [46]	Netherlands	2+2+0+1=5	15 (12-16) vs 15 (13-17)	64 vs 45	84 vs 86	Patients aged 10-19 years with IBD in clinical remission at baseline Patients diagnosed as having IBD more than 6 months before enrolment	Telemonitoring through web-based applications, email, and phone calls vs usual care	13	IBD-specific QoL Remission rate
Hunt et al, 2017 [44]	United States	0+0+0+1=1	36 (10) (total participants)	20.6 (total participants)	32 vs 31	Patients at least 18 years old who self-reported a previous diagnosis of IBD, according to a medical professional’s feedback for IBD patients Patients with secondary irritable bowel syndrome or with a known psychological risk factor for poor health-related QoL in chronic gastrointestinal tract disorders	Tele-education through cognitive behavioral therapy delivered online vs usual care	1.5	IBD-specific QoL Disease activity Depression
Krier et al, 2011 [45]	United States	1+2+1+1=5	62.8 (11.5) vs 58.5 (9.6)	87 vs 68	15 vs 19	Patients with IBD who underwent 57 encounters in 9 months	Teleconsulting through real-time image vs standard care	9	Patient satisfaction
McCombie et al, 2020 [49]	New Zealand	2+2+0+1=5	35.2 (12.4) vs 34.3 (12.9)	52 vs 46	50 vs 50	Patients who were 16 years or older with confirmed IBD and who had at least 2 outpatient appointments and <3 disease flares in the past 12 months	Telemonitoring through smartphone apps vs standard care	12	IBD-specific QoL
McCombie et al, 2016 [50]	New Zealand	2+0+0+1=3	38.3 (12.8) vs 39.6 (11.8)	33.6 vs 38.4	113 vs 86	All adults with IBD aged 18 to 65 years	Tele-education through computerized cognitive behavioral therapy vs usual care	6	IBD-specific QoL Generic QoL Depression
Miloh et al, 2017 [43]	United States	1+1+1+0=3	—	—	21 vs 30	Children with IBD who were 8 years and older	Telemonitoring through SMS text messaging vs standard care	12	Medication adherence rate Disease activity Number of clinic visits per patient
Ozgur Soy et al, 2019 [13]	Turkey	2+2+2+1=7	37.26(12.99) vs 41.63(11.85)	56.7 vs 60	30 vs 30	Adults aged 18 years or over who were diagnosed as having IBD for 6 months	Tele-education through web-based applications on the computer or phone vs standard care	2	IBD-specific QoL Remission rate
Schliep et al, 2020^d^ [41]	United States	2+2+0+1=5	37.3 (11.6) vs 39.3 (13.4) vs 39.5 (12.0)	45 vs 40.5 vs 37.5	71 vs 74 vs 72	Adults who were ≥18 years of age, were diagnosed as having IBD, and experienced at least one IBD flare in the 2 years prior to the baseline visit (an increase in IBD symptoms sufficient to warrant a change in medication dose or addition of a medication)	Telemonitoring and tele-education through a mobile phone with SMS text messaging vs standard care	12	Depressive symptoms Generic QoL

^a^These items were recorded as experimental vs control group.

^b^IBD: inflammatory bowel disease.

^c^QoL: quality of life.

^d^These studies came from the same clinical trial but reported different outcomes.

### Primary Outcomes

#### IBD-Specific Quality of Life

**Figure 4:**
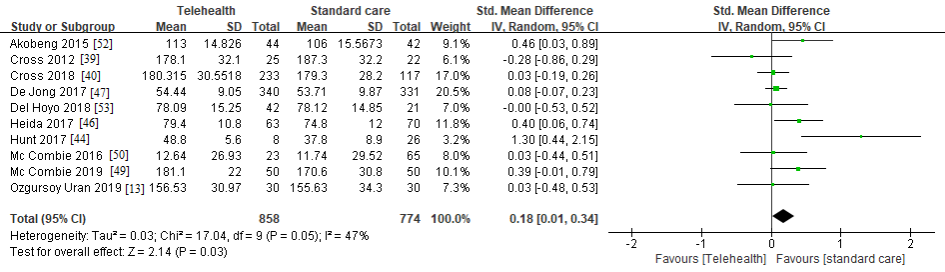
IBD-specific quality of life compared between telemedicine and standard care groups. IBD: inflammatory bowel disease [13,39,40,44,46,47,49,50,52,53].

A total of 10 clinical trials including 1632 participants were enrolled to compare IBD-specific QoL in the telemedicine and standard care groups. We found that IBDQ scores were higher in the telemedicine group than in the standard care group (SMD 0.18, 95% CI 0.01 to 0.34; *I^2^*=47; *P*=.03; Figure 4). Subgroup analysis stratified by follow-up time (<12 months or not) and participants characteristics (adults or adolescents) was conducted to examine the relatively high heterogeneity and identify the type of patients in need of telemedicine care. There was no significant difference in the IBDQ scores in the short-term (SMD 0.23, 95% CI −0.22 to 0.68; *I^2^*=61; *P*=0.31) or long-term subgroups (SMD 0.17, 95% CI 0 to 0.34; *I^2^*=47; *P*=.05; Multimedia Appendix 1). Furthermore, adolescents in the telemedicine group had significantly higher IBDQ scores than those in the standard care group (SMD 0.42, 95% CI 0.15 to 0.69; *I^2^*=0; *P*=.002), but no significant difference was found for adults between the groups (SMD 0.11, 95% CI −0.06 to 0.28; *I^2^*=41; *P*=.21; Multimedia Appendix 2).

**Figure 5:**
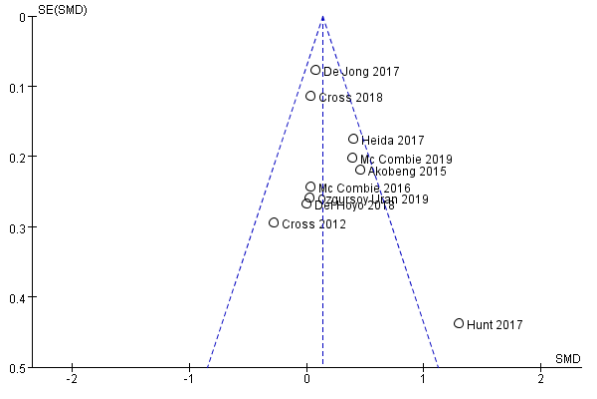
Funnel plot for potential publication bias. SMD: standard mean difference.

Funnel plot showed potential publication bias in our meta-analysis (Figure 5), contrary to the results of the Begg (*P*=.86) and Egger test (*P*=.26). This inconformity could be explained by the relatively small number of enrolled studies.

#### Disease Activity and Remission Rate

**Figure 6:**
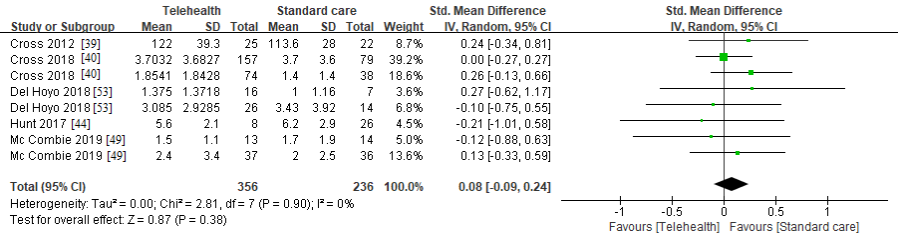
Disease activity in the telemedicine and standard care groups [39,40,44,53].

To examine the effectiveness of telemedicine in managing disease activity, 7 RCTs with a total of 955 patients were included. Disease activity was not significantly different between the telemedicine and standard care groups (SMD 0.08, 95% CI −0.09 to 0.24; *I^2^*=0; *P*=.38; Figure 6). Meanwhile, the remission rate in the telemedicine group was not significantly lower than that in the standard care group (relative risk 0.94, 95% CI, 0.83 to 1.05; *I^2^*=6; *P*=.26; Multimedia Appendix 3).

### Secondary Outcomes

#### Number of Clinic Visits per Patient

To investigate whether telemedicine intervention could lower the number of clinic or outpatient visits, we analyzed 6 articles that included 1479 patients with IBD. The number of clinic visits per patient was significantly lower in the telemedicine group than in the standard care group (SMD −0.71, 95% CI −1.07 to −0.36; *I^2^*=85; *P*<.001; Multimedia Appendix 4).

#### Patient Satisfaction

In 3 studies that included 183 participants, patient satisfaction was not significantly different between the telemedicine and standard care groups (SMD 0.21, 95% CI −0.12 to 0.54; *I^2^*=14; *P*=0.21; Multimedia Appendix 5).

#### Psychological Outcomes (Depression, General QoL, and Self-efficacy)

In the assessment of psychological outcomes, 7 clinical trials with 1165 participants showed no significant difference in the mental health of patients with IBD between the telemedicine and standard care groups (SMD −0.31, 95% CI −0.79 to 0.17; *I^2^*=66; *P*=.21 for depression score; SMD 1.37, 95% CI −0.42 to 3.15; *I^2^*=97; *P*=.13 for generic QoL; SMD 0.01, 95% CI −0.16 to 0.17, *I^2^*=23%; *P*=.95 for self-efficacy; Multimedia Appendices 6-8).

#### Medication Compliance

A total of 5 RCTs with 1169 patients with IBD were included to assess medication compliance. Considering that some articles reported the Morisky Scale score while others merely reported the medication compliance rate, we pooled data into 2 measures (medication compliance score and rate). Medication compliance in the telemedicine group did not improve significantly compared with that in the standard care group in terms of medication compliance score and rate (SMD 0.11, 95% CI −0.09 to 0.30; *I^2^*=19; *P*=.27 and relative risk 1.29, 95% CI 0.77 to 2.17; *I^2^*=88; *P*=.33, respectively; Multimedia Appendix 9).

## Discussion

There is high demand for long-term personalized care and medication to maintain remission and reduce the risk of relapse in patients with IBD [5,54]. Because of the convenience of use, telemedicine intervention may play an increasingly important role in managing IBD [27,55]. We aimed to investigate whether patients with IBD could benefit from telemedicine technology by performing a systematic review and meta-analysis. It was evident that enrolled studies exhibited obvious heterogeneity in the specific intervention used. The reason for this heterogeneity could not be identified because of the diversity and physical limitations of the IBD centers delivering telemedicine and their purposes and areas of application. However, regardless of the heterogeneity, we did find that patients who received telemedicine intervention had higher IBDQ scores and a significantly lower number of clinic visits per patient than those who received standard care. Importantly, adolescent patients with IBD benefit more from telemedicine and had significantly higher IBDQ scores that those who received standard care.

One possible reason is that there are more opportunities for the youth to access this relatively new form of care via the internet or mobile phones. Unlike in other chronic diseases (eg, chronic obstructive pulmonary disease), the peak onset of IBD is seen at a younger age [3,56]. This implies that telemedicine would be more acceptable in such patients with IBD. In addition, it seemed obvious that telemedicine could decrease the number of in-person clinic visits compared with standard care. However, none of the studies reported exact data or definitive conclusions on this issue. Considerable time and cost could be saved through the reduction of travel and waiting hours for regular office visits.

Given the robust effects of relapse or disease course on the daily life of patients with IBD, attention should be focused on relieving the psychological burden on these patients [57]. It is necessary to note that telemedicine aiming to improve outcomes in patients with IBD, such as through the incorporation of impactful web-based cognitive behavioral therapy (a form of tele-education), has proven to be an effective method for the management of mental health in patients with chronic gastrointestinal tract diseases [25]. However, our study showed no significant differences in psychological outcomes, such as depression, generic QoL, and self-efficacy, between the telemedicine and standard care groups. One potential explanation is that standard care provided by the referral center had built in emphasis on the importance of mental health care.

Theoretically, patients receiving telemedicine intervention have more access to report a flare than conventional follow-up and therefore receive more prompt consultation from health care givers. However, no significant differences were observed for disease activity and remission rates between these 2 groups in our meta-analysis. The reasons for this may be as follows: most patients were in remission at baseline, which led to a ceiling effect; it remains uncertain whether eHealth technologies could better influence the natural course of IBD compared with standard care; and evaluation of disease activity was based on the score self-reported by patients or their families without objective measurements. Hence, it is difficult to conclusively state the impact of telemedicine intervention on disease activity in patients with IBD.

In terms of therapeutic compliance, medication adherence was adequate in only around 45% of patients with IBD [58]. Nonadherence to medical therapy could cause a 5-fold increase in the risk of relapse, and low medication compliance correlates with lower QoL and higher cost of hospitalizations [59,60]. Thus, there is an urgent need to promote better medication compliance in patients with IBD. Our findings did not show a significant improvement in medication compliance in the telemedicine group, which was inconsistent with the outcomes reported by Rohde et al [26]. It is reasonable to speculate that this may be related to the compliance rate at baseline, as noncompliant patients might be more reluctant to participate in RCTs. Consequently, the participants enrolled are more compliant with the medication, which results in a ceiling effect.

Because of the superiority and popularity of mobile technology, intervention restricted to mobile phones is considered to be promising for the management of chronic diseases [61,62]. Our meta-analysis not only investigated the effectiveness of telemedicine in IBD, but also focused on the specific type of telemedicine, including mobile technology. All enrolled studies in our meta-analysis incorporated mobile devices into the telemedicine intervention, except 2 in which the intervention was confined to a computerized web-based system [45,50]. Therefore, we anticipate that our findings of the use of telemedicine for the management of IBD could also be applied to mobile technology.

Despite the strengths of this meta-analysis, there are certain limitations. First, there was an unavoidable high attrition rate in some studies that used the per protocol analysis. Second, some RCTs did not use the blinded-method design because of intervention characteristic limitations, which led to performance and detection biases. Third, the number of enrolled studies in the meta-analysis was relatively modest, which led to the contradictory results ascribed to potential publication bias. Finally, the specific population that would most benefit from telemedicine could not be identified because of a lack of complete reported data in the included studies.

In conclusion**,** constrained by the current limited material to provide telemedicine for IBD patients, the heterogeneity of specific telemedicine intervention was obviously evident. However, in accordance with the idea of providing health care from a distance, telemedicine should not be regarded as a uniform therapeutic method as is done for drug treatments but as a mode of health care delivery and even as an important adjuvant to routine clinical practice. Meanwhile, telemedicine intervention did show a promising role in improving IBDQ scores among adolescents and decreased the number of clinic visits by patients with IBD. Further research is needed to identify the patients with IBD who would most benefit from telemedicine.

## Appendix

Multimedia Appendix 1 Subgroup analysis of the inflammatory bowel disease questionnaire comparing the telemedicine with standard care by follow-up time.

Multimedia Appendix 2 Subgroup analysis of the inflammatory bowel disease questionnaire comparing the telemedicine with standard care by the age of patients.

Multimedia Appendix 3 Remission rate comparing the telemedicine versus the standard care.

Multimedia Appendix 4 The number of clinic visits per patient comparing telemedicine with standard care.

Multimedia Appendix 5 Patient satisfaction comparing telemedicine with standard care.

Multimedia Appendix 6 Depression comparing the telemedicine with the standard care.

Multimedia Appendix 7 Generic quality of life comparing the telemedicine with the standard care.

Multimedia Appendix 8 Self-efficacy comparing the telemedicine with the standard care.

Multimedia Appendix 9 (A) Medication compliance score comparing the telemedicine with the standard care.(B) Medication compliance rate comparing the telemedicine with the standard care.

## Abbreviations

IBD: inflammatory bowel disease
IBDQ: inflammatory bowel disease questionnaire
PRISMA: Preferred Reporting Items for Systematic Reviews and Meta-Analyses
QoL: quality of life
RCT: randomized controlled trial
