# Supplementary figures and images for: Role of Telemedicine in Inflammatory Bowel Disease: Systematic Review and Meta-analysis of Randomized Controlled Trials
Source: J Med Internet Res. 2022 Mar 24;24(3):e28978. doi: 10.2196/28978 (PMC8990345; doi:10.2196/28978)

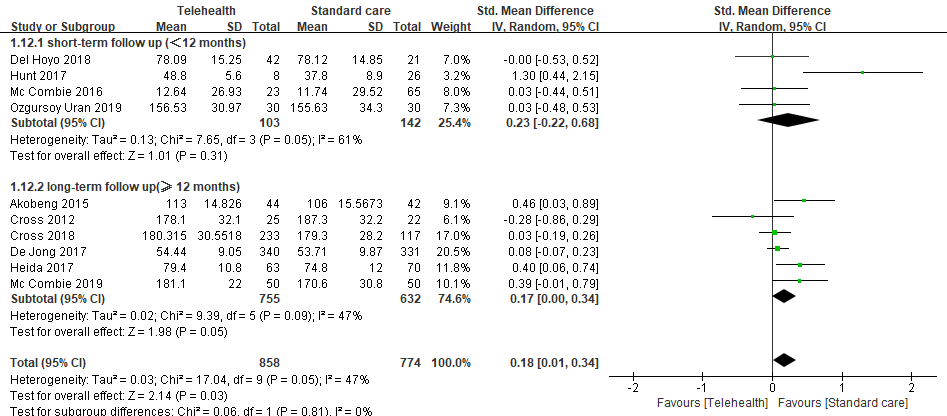

Supplement: Multimedia Appendix 1 [file jmir_v24i3e28978_app1.png]

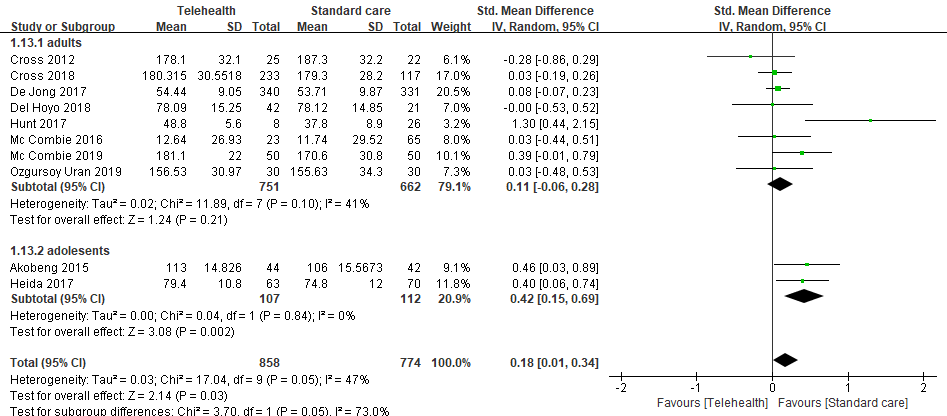

Supplement: Multimedia Appendix 2 [file jmir_v24i3e28978_app2.png]

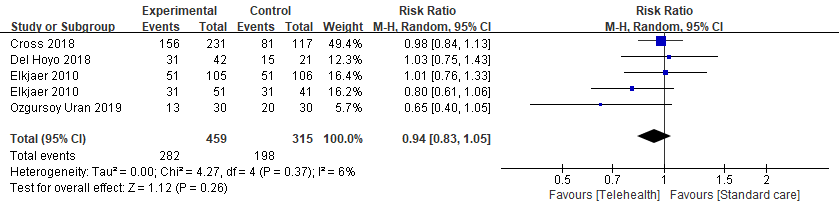

Supplement: Multimedia Appendix 3 [file jmir_v24i3e28978_app3.png]

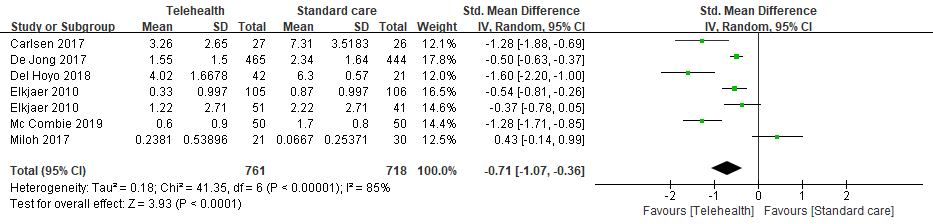

Supplement: Multimedia Appendix 4 [file jmir_v24i3e28978_app4.png]

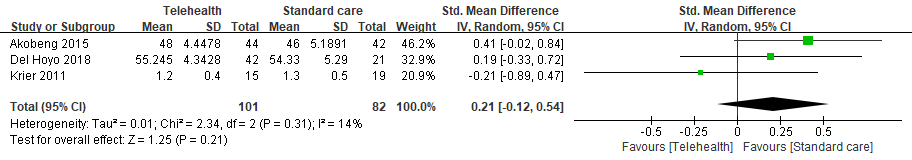

Supplement: Multimedia Appendix 5 [file jmir_v24i3e28978_app5.png]

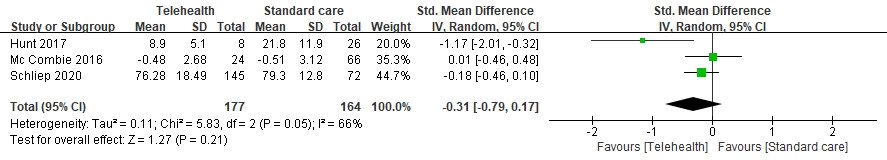

Supplement: Multimedia Appendix 6 [file jmir_v24i3e28978_app6.png]

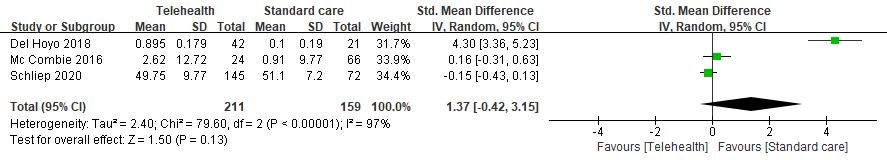

Supplement: Multimedia Appendix 7 [file jmir_v24i3e28978_app7.png]

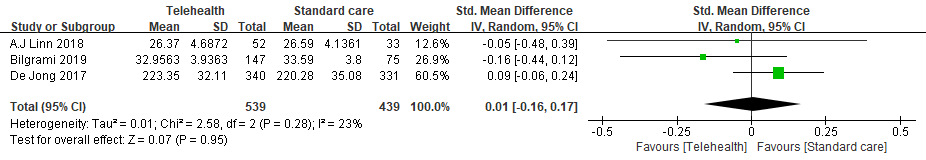

Supplement: Multimedia Appendix 8 [file jmir_v24i3e28978_app8.png]

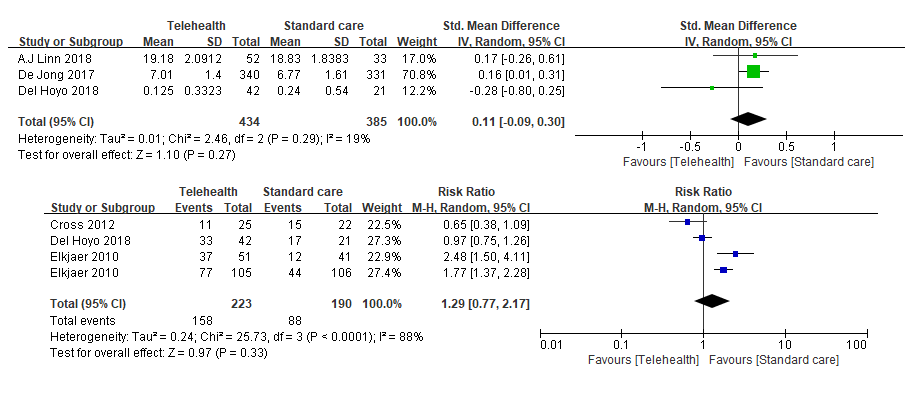

Supplement: Multimedia Appendix 9 [file jmir_v24i3e28978_app9.png]
